# Effect of Liver Fibrosis on Oral and Gut Microbiota in the Japanese General Population Determined by Evaluating the FibroScan–Aspartate Aminotransferase Score

**DOI:** 10.3390/ijms241713470

**Published:** 2023-08-30

**Authors:** Satoshi Sato, Chikara Iino, Daisuke Chinda, Takafumi Sasada, Tetsuyuki Tateda, Masatoshi Kaizuka, Hiroki Nomiya, Go Igarashi, Kaori Sawada, Tatsuya Mikami, Shigeyuki Nakaji, Hirotake Sakuraba, Shinsaku Fukuda

**Affiliations:** 1 Department of Gastroenterology and Hematology, Hirosaki University Graduate School of Medicine, Hirosaki 036-8562, Japan; satoshis@hirosaki-u.ac.jp (S.S.); ssd.tkfm@hirosaki-u.ac.jp (T.S.); t.tateda@hirosaki-u.ac.jp (T.T.); m.kaizuka@hirosaki-u.ac.jp (M.K.); h.nomiya@hirosaki-u.ac.jp (H.N.); iga0910@hirosaki-u.ac.jp (G.I.); hirotake@hirosaki-u.ac.jp (H.S.); sfukuda@hirosaki-u.ac.jp (S.F.); 2 Division of Endoscopy, Hirosaki University Graduate School of Medicine, Hirosaki 036-8562, Japan; 3 Department of Preemptive Medicine, Hirosaki University Graduate School of Medicine, Hirosaki 036-8562, Japan; iwane@hirosaki-u.ac.jp (K.S.); tmika@hirosaki-u.ac.jp (T.M.); 4 Center of Healthy Aging Innovation, Hirosaki University Graduate School of Medicine, Hirosaki 036-8562, Japan; nakaji@hirosaki-u.ac.jp

**Keywords:** liver fibrosis, oral microbiota, gut microbiota, FibroScan–asparate aminotransferase (FAST) score

## Abstract

The association between liver fibrosis and oral or gut microbiota has been studied before. However, epidemiological studies in the general population are limited owing to the difficulty of noninvasive liver-fibrosis assessment. FibroScan–asparate aminotransferase (FAST) scores can be used to accurately and non-invasively evaluate liver fibrosis. This study aimed to determine the association between liver fibrosis and oral or gut microbiota using the FAST score in the general population. After propensity score matching of 1059 participants based on sex, age, body mass index, homeostasis model assessment of insulin resistance, and triglyceride levels, 125 (non-liver-fibrosis group, 100; liver fibrosis group, 25) were included. The diversity of gut microbiota differed significantly between the two groups; however, no significant differences were noted in their oral microbiota. The liver fibrosis group showed an increase in the relative abundance of *Fusobacteria* strains and a decrease in the relative abundance of *Faecalibacterium*, with the presence of *Fusicatenibacter* in the gut microbiota. *Feacalibacterium* was not identified as an independent factor of liver fibrosis in adjusting the fatty liver index. In the general population, gut microbiota may be more involved in liver fibrosis than oral microbiota.

## 1. Introduction

Alcohol consumption, hepatitis B and C, and metabolic dysfunction-associated steatotic liver disease (MASLD) can lead to advanced stages of liver fibrosis, which can progress to cirrhosis and is a risk factor for liver cancer. In many liver diseases, the degree of fibrosis is strongly related to patient prognosis, including overall mortality [1,2].

Chronic liver diseases due to alcohol consumption, hepatitis B and C, and MASLD cause dysbiosis of the gut microbiota from their early stages [3,4,5,6]. Gut dysbiosis disrupts the intestinal barrier, causing bacterial translocation and the entry of pathogenic bacteria and pathogen-associated molecular patterns (e.g., lipopolysaccharide (LPS)) into the portal vein. This leads to hepatic inflammation cascades, which further result in fibrosis and other diseases. The relationship between the gut microbiota and the liver is termed the gut–liver axis [7,8], and the association between gut microbiota and liver fibrosis has been studied extensively. Patients with liver fibrosis can reportedly develop gut dysbiosis, with an increase in Streptococcus, Proteobacteria, and Fusobacteria strains and a decrease in *Ruminococcaceae* and *Lachnospiraceae* strains, regardless of the cause of hepatitis [9,10,11,12,13,14,15,16]. Bajaj et al., observed that the ratio of beneficial taxa (*Lachnospiraceae* + *Ruminococcaceae* + *Veillonellacea* + *Clostridiales Incertae Sedis XIV*) to pathogenic taxa (*Enterobacteriaceae* + *Bacteroidaceae*) was lower in patients with liver cirrhosis than in healthy individuals [17]. Additionally, oxidative stress has also been implicated in the relationship between liver fibrosis and gut microbiota. Excessive oxidative stress causes liver fibrosis through the production of inflammatory cytokines [18,19]. In a vicious cycle, the consequent development of liver fibrosis further increases oxidative stress. Alcohol consumption, hepatitis B and C, NAFLD, and many other chronic liver diseases increase oxidative stress through iron overloading, increased cytokine levels, and endotoxemia [20,21,22,23,24,25]. Of these, the endotoxins are internalized in the cell walls of gram-negative bacteria, and dysbiosis results in increased blood levels of endotoxins. Therefore, dysbiosis promotes liver fibrosis due to increased oxidative stress.

Recently, the association between chronic liver diseases and oral microbiota has also received attention. Several studies have implicated oral dysbiosis in chronic liver diseases. Some gut bacteria associated with liver fibrosis are known to migrate from the oral cavity [26,27,28]. Furthermore, oral *Porphyromanas gingivalis*, the key pathogen in chronic periodontitis, can reportedly reach the liver hematogenously and induce liver fibrosis [29,30,31]. Therefore, oral and gut microbiota have interrelated or independent effects on the development of liver fibrosis.

Previous studies have used invasive liver biopsy to evaluate liver fibrosis. Recently, FibroScan has enabled the noninvasive evaluation of liver fibrosis. The FibroScan–aspartate aminotransferase (FAST) score is a simple algorithm developed to diagnose nonalcoholic steatohepatitis (NASH) with advanced fibrosis [32]. The FAST score is calculated using a formula that involves the liver stiffness measurement (LSM) and controlled attenuation parameter (CAP), which are measured using FibroScan and blood aspartate transaminase, allowing precise evaluation of liver fibrosis without liver biopsy. AST levels in hepatocytes reflect the degree of hepatic inflammation because it is released into the blood when hepatocytes are damaged [33]. By including AST as a variable in the formula, the FAST score can provide a detailed assessment of systemic inflammation and oxidative stress related to liver fibrosis. Although several non-invasive liver fibrosis assessment tools using FibroScan have been introduced, the FAST score has been shown to be the most reliable [34]. The FAST score was developed for the diagnosis of NASH; however, its utility extends to the evaluation of metabolic-associated fatty liver disease (MAFLD) in the general population and follow-up of patients with hepatitis C after sustained virologic response [35,36]. Furthermore, the American Diabetes Association recommends LSM using FibroScan for patients with type 2 diabetes and elevated alanine aminotransferase (ALT) levels or fatty liver [37].

The association between liver fibrosis and oral or gut microbiota has been widely studied. However, few epidemiological studies have evaluated the general population owing to the challenges of noninvasive liver fibrosis assessment. Previous studies on the general population have also used tomographic ultrasonography to subjectively evaluate the liver [38,39]. Therefore, the evaluation methods of previous studies have varied, and their accuracy may be insufficient.

This study aimed to investigate the association between liver fibrosis and oral or gut microbiota in the general population through a large epidemiological study while adjusting for confounding factors affecting liver fibrosis and oral or gut microbiota.

## 2. Results

### 2.1. Participant Characteristics

**Figure 1:**
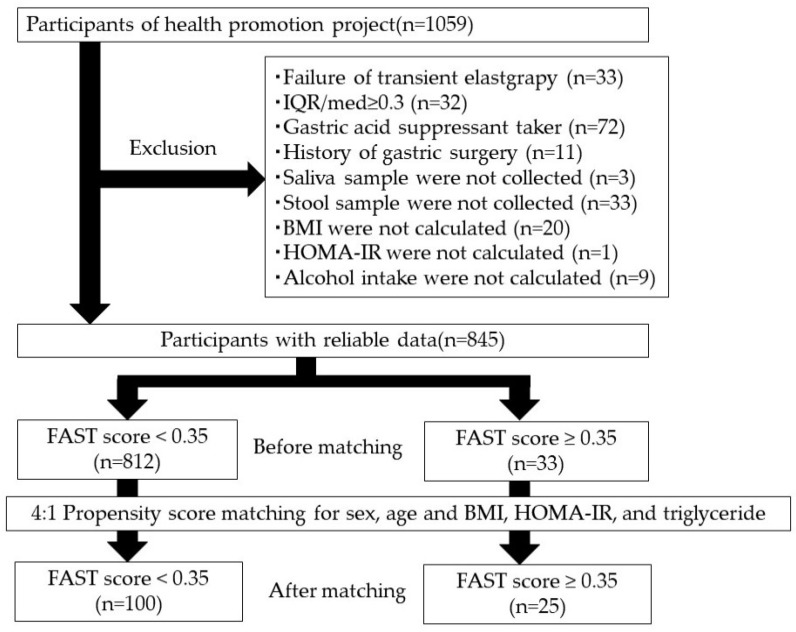
Flowchart for participant selection. For analysis based on liver fibrosis status, we selected 100 and 25 participants without and with liver fibrosis, respectively. The participants were categorized using propensity score matching. BMI, body mass index; FAST score, FibroScan–aspartate aminotransferase score; HOMA-IR, homeostasis model assessment of insulin resistance; IQR/med, interquartile range/median.

The study flowchart is presented in Figure 1, and the baseline participant characteristics are shown in Supplementary Table S1. Of the screened participants with reliable data, 238 (28.2%) were diagnosed with MAFLD, 223 (26.4%) with MASLD, 46 (5.4%) were heavy alcohol drinkers, 8 (0.9%) were hepatitis B surface (HBs) antigen positive, and 16 (1.9%) were hepatitis C virus (HCV) antibody positive. Further, 33 (3.9%; 21 males and 12 females) of them had a FAST score ≥ 0.35. Among patients with MAFLD, MASLD, and heavy alcohol drinking, 24 (10.1%), 24 (10.8%), and 4 (8.7%) had a FAST score ≥ 0.35, respectively. Significant differences were observed in the sex, age, body mass index (BMI), homeostasis model assessment of insulin resistance (HOMA-IR), and triglyceride of participants with FAST scores < 0.35 and ≥ 0.35.

Following propensity score matching, the two groups showed significant differences in the aspartate aminotransferase, ALT, and gamma-glutamyl trans-peptidase levels and the fibrosis-4 (FIB-4) and fatty liver indices. MASLD was significantly more common in the group with FAST scores ≥ 0.35; however, no differences were observed in the prevalence of MAFLD, heavy alcohol consumption, HBs antigen positivity, and HCV antibody positivity between both groups (Table 1).

**Figure 2:**
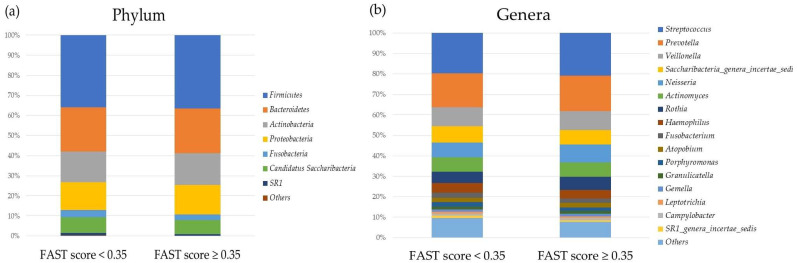
Relative abundances of the oral microbiota in the groups with FAST scores < 0.35 and ≥0.35 at (**a**) the phylum and (**b**) genus levels.

**Figure 3:**
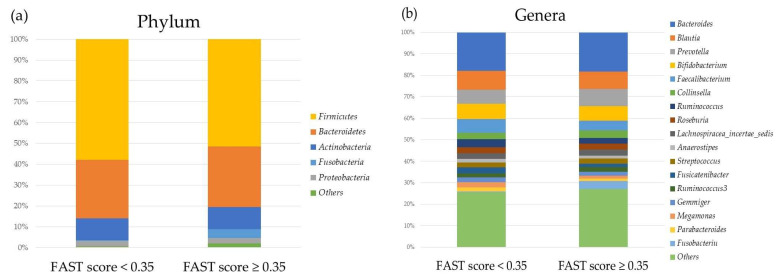
Relative abundances of gut microbiota between the groups with FAST scores < 0.35 and ≥ 0.35 at (**a**) the phylum and (**b**) genus levels.

Figure 2 and Figure 3 show differences in the relative abundances of oral and gut microbiota between the groups with FAST scores < 0.35 and ≥0.35 at the phylum and genus levels, with relative abundances of more than 1% after propensity score matching.

**Figure 4:**
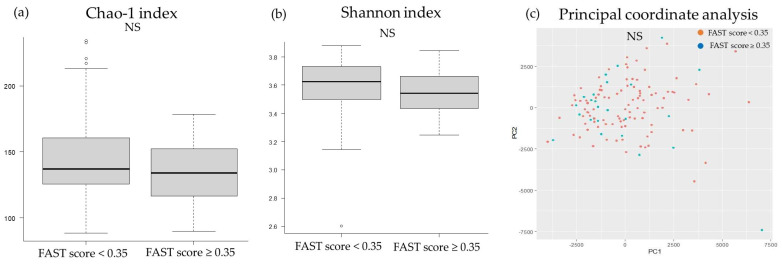
Comparison of the diversity of oral microbiota and liver fibrosis: (**a**) chao-1 index, (**b**) Shannon index, (**c**) principal coordinate analysis. FAST score, FibroScan–aspartate aminotransferase score; NS, not significant; pc, principal components.

**Figure 5:**
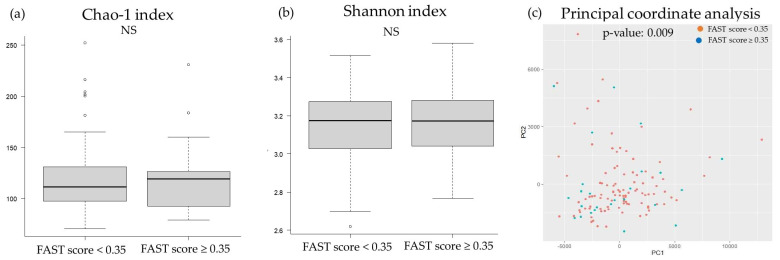
Comparison of the diversity of gut microbiota and liver fibrosis: (**a**) chao-1 index, (**b**) Shannon index, (**c**) principal coordinate analysis. FAST score, FibroScan–aspartate aminotransferase score; NS, not significant; pc, principal component.

The two groups showed no significant difference in the chao-1 index, Shannon index, and principal coordinate analysis results for the oral microbiota (Figure 4). On the contrary, the principal coordinate analysis for the gut microbiota revealed microbial structural differences between the groups, although there were no significant differences in the chao-1 and Shannon indices (Figure 5).

### 2.2. Comparison of the Oral and Gut Microbiota and Liver Fibrosis

**Figure 6:**
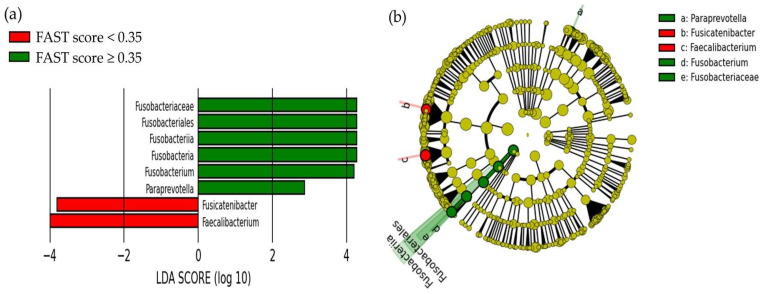
Linear discriminant analysis effect size results of the gut microbiota for the groups with FAST scores < 0.35 and ≥ 0.35: (**a**) the linear discriminant, and (**b**) cladogram report. FAST score, FibroScan–aspartate aminotransferase score; LDA score, linear discriminant analysis score.

**Figure 7:**
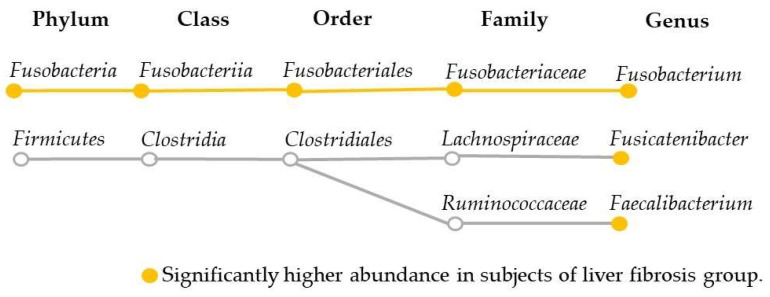
Systematic diagram of gut bacteria with ≥1% relative abundance.

The linear discriminant analysis effect size (LEfSe) results of liver fibrosis and oral or gut microbiota after propensity score matching are shown in Figure 6. The groups with FAST scores < 0.35 and ≥0.35 showed differences in gut microbiota (Figure 6). In the group with a FAST score ≥ 0.35, we observed an increase and decrease in six and two gut bacterial species, respectively. Of these gut microbiota, those with a relative abundance of ≥1% were *Fusobacteria* (1.2%), *Fusobacteriia* (1.2%), *Fusobacteriales* (1.2%), *Fusobacteriaceae* (1.2%), and *Fusobacterium* (1.1%) in the increased group, and *Faecalibacterium* (6.0%) and *Fusicatenibacter* (2.6%) in the decreased group (Figure 7). The relative abundance of *Paraprevotella* was negligible at 0.002%. On the contrary, no difference was noted in the oral bacterial species of both groups.

### 2.3. Correlation between FAST score, FIB-4 Index, Fatty Liver Index, and Gut Microbiota

Correlations between the FAST score, FIB-4 index, fatty liver index, and gut bacteria with high relative abundance are shown in Table 2. There were positive significant correlations between the FAST score and *Fusobacteria* strains and negative significant correlations between the FAST score and *Feacalibacterium*. On the contrary, no significant correlation was observed between the FIB-4 index and gut microbiota. Only *Feacalibacterium* showed a negative correlation with the fatty liver index.

### 2.4. Risk Factors for Liver Fibrosis Adjusted by Fatty Liver Index

The results of the multivariate analysis for risk factors of liver fibrosis, after adjusting for the fatty liver index to exclude the effect of steatosis, are shown in Table 3. The increase in the *Fusobacterium* population and decrease in the *Fusicatenibacter* population were independently associated with liver fibrosis. However, *Feacalibacterium* was not identified as an independent factor of liver fibrosis on adjusting for the fatty liver index.

## 3. Discussion

This study of the general population using the FAST score revealed an increase in the relative abundance of *Fusobacteria* strains and decrease in the relative abundance of *Faecalibacterium* and *Fusicatenibacter* in the gut microbiota of participants with liver fibrosis. Moreover, the multivariate analysis showed that *Fusobacterium* and *Fusicatenibacter* were significant factors in the development of liver fibrosis after adjustment for fatty liver index. On the contrary, the oral microbiota associated with liver fibrosis could not be identified.

The association between liver fibrosis and gut microbiota is known as the gut–liver axis; however, the association between liver fibrosis and oral microbiota remains unclear. Nonetheless, oral dysbiosis has been implicated in patients with chronic liver diseases, and some gut bacteria associated with liver fibrosis have been shown to migrate from oral microbiota [26,27,28]. In addition, chronic hepatitis, such as viral hepatitis, has been reported to decrease oral diversity [27,40]. In the present study, with regard to oral microbiota, no significant differences were observed between the non-liver fibrosis and liver fibrosis groups in both alpha and beta diversity. The liver fibrosis group in this study comprised more cases of MASLD and fewer cases of heavy alcohol consumption and viral hepatitis, which may explain the differences in the results of the present and previous studies. On the contrary, with regard to the gut microbiota, there was no difference in the alpha diversity between the non-liver fibrosis and liver fibrosis groups, although there was a significant difference in beta diversity. Liver fibrosis due to alcohol, viral hepatitis, and autoimmune hepatitis, such as primary biliary cholangitis, decreases both alpha and beta diversities [41,42,43,44]. Furthermore, it has been reported that the beta diversity in patients with NASH or MASLD differs from that in healthy controls, although the alpha diversity appears unchanged [45,46]. As most patients in the liver fibrosis group in this study had MASLD, with few cases of heavy alcohol consumption, HBs antigen positivity, and HCV antibody positivity, our results were generally consistent with those of previous studies on NASH or MASLD.

This study revealed that the gut relative abundance of *Fusobacteria* strains, including *Fusobacteria*, *Fusobacteriia*, *Fusobacteriales*, *Fusobacteriaceae*, and *Fusobacterium,* was increased in the liver fibrosis group. Gram-negative bacteria, which contain LPS (an endotoxin) internalized in the cell wall, are increased in patients with cirrhosis [47]. They reach the liver and stimulate the hepatic inflammation cascades, leading to fibrosis and other diseases. Inflammation also occurs when the endotoxin of Gram-negative bacteria reaches the liver hematogenously owing to impaired gut barrier function. In patients with liver disease, the serum levels of endotoxins, such as LPS, increase in the blood [48]. LPS is a component of the outer extracellular membrane of intestinal Gram-negative bacteria and reaches the liver via the portal vein due to a decrease in the intestinal permeability caused by increased Gram-negative bacteria and disruption of intestinal barrier function. When LPS reaches the liver, tumor growth factor-β signal is activated through suppression of bone morphogenetic protein and activin membrane-bound inhibitor expression in hepatic stellate cells via toll-like receptor 4, and liver fibrosis is promoted [49]. Endotoxins also increase oxidative stress through nicotinamide adenine dinucleotide phosphate oxidase 2 and 8-iso-prostaglandin F2α, further promoting liver fibrosis [50,51]. *Fusobacteria* strains, which were increased in the high FAST score group in this study, are representative of Gram-negative bacteria producing LSP. Therefore, the increase in the relative abundance of *Fusobacteria* strains may lead to an increase in the serum level of LPS and thereby cause liver fibrosis.

In this study, the gut relative abundance of *Faecalibacterium* and *Fusicatenibacter*, which are considered the major butyric acid-producing bacteria, was decreased in the liver fibrosis group. Butyric acid, a short-chain fatty acid, is reportedly fermented by gut microbiota on dietary fiber substrate and is decreased in cirrhosis [47]. Bajaj et al., reported that *Ruminococcaceae* and *Lachnospiraceae* have similar abilities in reducing butyric acid in patients with cirrhosis [10]. Butyric acid suppresses intestinal permeability and inflammation via regulatory T cells, thereby reducing the influx of toxic substances, including endotoxin into the liver [47,52]. Furthermore, butyric acid has been reported to protect against oxidative stress [53,54,55]. In mice, administration of butyrate-producing bacteria showed a hepatoprotective effect for acute liver damage [53]. *Faecalibacterium* is the most abundant butyric acid-producing bacteria, and its levels are reduced during inflammatory bowel disease and MASLD [39,56,57]. Our findings suggest that butyric acid levels may have decreased due to the reduction in *Faecalibacterium* and *Fusicatenibacter* in the gut of the liver fibrosis group.

We performed propensity score matching based on HOMA-IR and triglyceride levels in addition to sex, age, and BMI to equalize the background characteristics of the non-liver-fibrosis and liver-fibrosis groups. Insulin resistance and dyslipidemia influence the development of liver fibrosis, including NASH [58,59,60]. Furthermore, an increase in *Fusobacterium* levels and a decrease in *Faecalibacterium* levels were observed in patients with diabetes [61]. In this study, the HOMA-IR and triglyceride levels were significantly higher in the liver fibrosis group before propensity score matching. Therefore, we matched both items to adjust for their effects before comparison of the groups. Owing to the anti-inflammatory effects of butyric acid, which were due to a protective effect against intestinal permeability, a negative correlation between *Faecalibacterium* levels and insulin resistance was observed [62]. On the contrary, LPS produced by Gram-negative bacteria, such as *Fusobacterium,* has been reported to exacerbate insulin resistance by inducing inflammation [63]. The present results suggest that *Fusobacteria* strains, *Feacalibacterium*, and *Fusicatenibacter* may induce the development of liver fibrosis via mechanisms other than insulin resistance and dyslipidemia. On the contrary, *Feacalibacterium* was not revealed as an independent factor in adjusting for the fatty liver index. *Feacalibacterium* has been reported to significantly reduce in patients with MASLD even in the absence of fibrosis [49]. In addition, most participants in the liver-fibrosis group had MASLD. *Feacalibacterium* might be relatively more affected by liver fat mass than by liver fibrosis.

Further, no correlation was observed between the FIB-4 index, a representative liver fibrosis index, and gut bacterial species as revealed by the FAST score. The FIB-4 index is useful and can be easily calculated, but it does not reflect the condition of the liver. In addition, because age is considered in the formula for the FIB-4 index, the score is naturally higher for elderly individuals. Further, gut microbiota is greatly affected by aging. Therefore, although the FIB-4 index was significantly higher in the liver-fibrosis group after propensity score matching in this study, the association may not have been evident owing to the above reasons.

In previous studies, gut *Streptococcaceae* and *Enterobacteriaceae* have been reported to increase in liver fibrosis [9,10]. However, no association between liver fibrosis and these bacteria was observed in the present study. This discrepancy may be attributable to the differences in the target population of the two studies. Although previous studies have included patients who were visiting or admitted to the hospital for chronic hepatitis or cirrhosis, the current study included a general population who underwent a medical checkup. In addition, gastric acid suppressants, such as proton pump inhibitors, are often prescribed for patients with cirrhosis and disrupt gut microbiota [64,65]. Oral indigenous bacteria, such as *Streptococcaceae,* reach and increase in the gut without being sterilized by gastric acid [66]. In the current study, we excluded patients with decreased gastric acid secretion, such as those taking gastric suppressants or with a history of gastric surgery, to exclude the effect of gastric acid on gut microbiota.

Our study did not reveal the oral bacterial species associated with liver fibrosis. Oral *Porphyromanas gingivalis*, the main causative agent of chronic periodontitis, has been reported to be involved in the pathological progression of NASH [29,30,31]. In fatty liver, toll-like receptor 2 expression is increased in hepatocytes, and the sensitivity to LPS and plasma membrane components of *Porphyromanas gingivalis* is also increased, resulting in excessive cytokine production, which has a significant influence on NASH pathogenesis [30]. Furthermore, the enzyme gingipain produced by *Porphyromanas gingivalis* promotes the production of transforming growth factor-β and aggravates liver fibrosis [31]. In addition, some strains of gut *Fusobacteria* have been reported to be the same as oral Fusobacteria strains [62,67]. In this study, no association was observed between oral *Fusobacteria* and liver fibrosis, although gut *Fusobacteria* were associated with liver fibrosis. In previous studies, NASH was diagnosed using tissues from liver biopsies, and the discrepant findings may be attributed to these methodological differences.

This study has several limitations. First, given that this study involved an examination of the general population, the number of participants with a FAST score ≥ 0.35 was small. A larger sample size may yield different and new findings in the future. Second, the diagnosis of liver fibrosis was made using FibroScan and not liver biopsy, which is an invasive procedure. It was not possible to perform a liver biopsy for the general population. However, the FAST score was used to establish the diagnosis of liver fibrosis. Third, the rates of MASLD and MAFLD among the study participants were high, with low values of the BMI and other parameters associated with cardiovascular risk. Although obesity and MASLD are strongly associated, nonobese fatty liver is common in Asia; therefore, this finding is not generalizable to all ethnicities [68,69]. Fourth, we did not measure the relevant markers of oxidative stress, which plays an important role in liver fibrosis. Oxidative stress is a major contributor to endotoxin-mediated liver fibrosis caused by dysbiosis. In this study, the addition of oxidative stress-related markers to the study of the association between liver fibrosis and oral or gut microbiota could have provided important insights.

## 4. Materials and Methods

### 4.1. Study Participants

In total, 1059 individuals who participated in the Iwaki Health Promotion Project Health Survey held in June 2018 in Aomori Prefecture, northern Japan, were invited to participate in this study (Figure 1). This study was performed in accordance with the ethical standards of the Declaration of Helsinki and was approved by the Medical Ethics Committee of Hirosaki University (authorization number: 2018-063). Written informed consent was obtained from all participants. Of them, 214 participants who had a failure of transient elastography measurement, were taking gastric acid secretion inhibitors, and had a history of gastric surgery did not have saliva or stool sample or BMI data, did not have HOMA-IR, and alcohol intake data were excluded. Participants were assigned to the non-liver-fibrosis and liver-fibrosis groups based on their FAST scores. To equalize the background characteristics of both groups, 4:1 propensity score matching was performed with sex, age, BMI, HOMA-IR, and triglyceride levels, all of which influence gut microbiota.

### 4.2. Transient Elastography

Transient elastography with LSM and CAP measurements was performed using a FibroScan 530 compact device (Echosens, Paris, France) equipped with both M and KL probes. The examinations were performed by five well-trained hepatology specialists. When the number of measurements was less than 10 or the ratio of the interquartile range was greater than 0.30, the measured values were excluded because of unreliability.

### 4.3. Clinical Parameters

The following clinical parameters were recorded on the same day as the transient examination: sex; age; height; BMI (calculated by dividing the weight in kilograms by the squared height in meters); results for HBs antigen or anti-HCV test; and levels of aspartate aminotransferase, alanine aminotransferase, gamma-glutamyl transpeptidase, albumin, total bilirubin, glucose, insulin, hemoglobin A1c (HbA1c), high-density lipoprotein cholesterol, low-density lipoprotein cholesterol, triglycerides, platelets, and C-reactive protein. The insulin resistance index was calculated using HOMA-IR as follows: fasting glucose (mg/dL) × fasting insulin (µU/mL)/405. The liver fibrosis index other than the FAST score was calculated using the FIB-4 index as follows:

{age × AST (U/L)}/{blood platelet count (109/L) × √ALT (U/L)]}.

The fatty liver index was calculated as follows:

{e(0.953 × ln(triglycerides) + 0.139 × BMI + 0.718 × ln(γ-GTP) + 0.053 × WC − 15.745)}/{1 + e (0.953 × ln(triglycerides) + 0.139 × BMI + 0.718 × ln(γ-GTP) + 0.053 × WC − 15.745)} × 100.

On the basis of a previous report, participants with a fatty liver according to a CAP of 248 dB/m or higher who met any of the following criteria were diagnosed with MAFLD: obesity (BMI ≥ 23 kg/m^2^); type 2 diabetes; or BMI < 23 kg/m^2^ with two or more metabolic dysregulations (waist circumference ≥ 90 cm and ≥80 cm in males and females, respectively; blood pressure ≥ 130/85 mmHg or specific drug treatments; triglyceride levels ≥ 150 mg/dL or specific drug treatment; impaired glucose tolerance [fasting blood glucose ≥ 100 mg/dL or HbA1c ≥ 5.7%]; HOMA-IR ≥ 2.5; and CRP level ≥ 2 mg/dL) [70]. MASLD was defined as a fatty liver on transient elastography in the absence of the following: excessive alcohol consumption (≥30 g/day in males and ≥20 g/day in females), HBs antigen or HCV antibody positivity, and use of steatogenic medications, such as amiodarone, methotrexate, corticosteroids, and tamoxifen.

### 4.4. FAST Score

The FAST score was developed by Newsome et al., in 2020 and is calculated as follows: {exp (–1.65 + 1.07 × ln (LSM) + 2.66 × 10 − 8 × CAP3 − 63.3 × AST − 1)}/{1 + exp (–1.65 + 1.07 × ln (LSM) + 2.66 × 10 − 8 × CAP3 − 63.3 × AST − 1)} [32]. The FAST score is the predicted probability from the logistic regression model. Therefore, it is bounded between zero and one and can be interpreted in a probabilistic manner. Newsome et al., reported a FAST score < 0.35 as a rule-out zone (sensitivity 90%, specificity 53%, negative predictive value 85%) and a FAST score ≥ 0.67 as a rule-in zone (sensitivity 48%, specificity 90%, positive predictive value 83%) [24]. In this study, a FAST score of 0.35, which is a rule-out zone, was used as the cutoff value.

### 4.5. Next-Generation Sequence Analysis of Oral and Gut Microbiota

Saliva and fecal samples were collected in commercial containers (TechnoSuruga Laboratory Co., Ltd., Shizuoka, Japan) and suspended in guanidine thiocyanate solution (100 mM Tris-HCL (pH 9.0), 40 mM Tris-EDTA (pH 8.0), 4M Guanidine Thiocyanate). These samples were kept at −80 °C prior to DNA extraction. According to previous studies, a series of representative bacterial species in the human gut microbiota were analyzed using primers for the V3–V4 region of 16S rDNA of prokaryotes. Sequencing was performed using an Illumina MiSeq system (Illumina, San Diego, CA, USA) [71]. The methods for quality filtering of the sequences were as follows: the only reads that had quality value scores for scores ≥ 0 for more than 99% of the sequences were extracted for the analysis. Detection and identification of the bacteria from the sequences were performed using Metagenome@KIN software (R-4.1.1. World Fusion Co., Tokyo, Japan) and the TechnoSuruga Lab Microbial Identification database DB-BA 10.0 (TechnoSuruga Laboratory, Shizuoka, Japan) at 97% sequence similarity. Relative abundance is presented as the percent composition of reads for each bacterium relative to the total number of reads.

### 4.6. Statistical Analysis

Categorical variables are presented as frequencies, whereas continuous variables are presented as medians with interquartile ranges. Comparisons between the two groups were made using χ-square and Mann–Whitney U tests for independence. Spearman’s rank correlation coefficients were calculated to determine the correlation between the FAST score and bacteria. Logistic regression analysis was used to perform multivariate analysis of binary variables. Statistical analyses of the clinical data were performed using the Statistical Package for the Social Sciences (SPSS) version 28.0 (SPSS Inc., Chicago, IL, USA) and R software (R Foundation for Statistical Computing, version R-4.1.1).

In the analysis of oral and gut microbiota, alpha diversity was evaluated using chao-1 and the Shannon index, and beta diversity was evaluated using principal coordinate analysis. To compare the non-liver-fibrosis and liver-fibrosis groups, LEfse 1.0 software was used [72].

To equalize the background factors of non-liver-fibrosis and liver-fibrosis groups, propensity score matching was performed with sex, age, BMI, HOMA-IR, and triglyceride levels. All analyses, except those described in Supplementary Table S1, were performed after the propensity score matching of participants.

A *p*-value <0.05 was considered to indicate statistical significance.

## 5. Conclusions

In summary, we found that an increase in *Fusobacteria* strains, and a decrease in *Faecalibacterium* and *Fusicatenibacter* in gut microbiota were significant factors in the development of liver fibrosis. On the contrary, we did not identify oral microbiota associated with liver fibrosis. In the general population, gut microbiota may be more involved in liver fibrosis than oral microbiota.

## Figures and Tables

**Table 1:** Participant characteristics after matching for sex, age, and BMI.

	FAST < 0.35 (n = 100)	FAST ≥ 0.35 (n = 25)	*p*-Value
Sex (male/female)	55:45	16:9	0.557
Age (years)	58.0 (45.8–68.0)	64.0 (44.0–68.0)	0.765
BMI (kg/m^2^)	25.3 (24.0–27.1)	25.0 (24.1–25.6)	0.412
AST (U/L)	21.0 (17.0–25.0)	46.0 (40.0–55.0)	<0.001
ALT (U/L)	20.0 (15.8–26.0)	63.0 (43.0–76.0)	<0.001
γGTP (U/L)	26.0 (18.0–40.0)	87.0 (53.0–116.0)	<0.001
HOMA-IR	1.36 (1.06–2.10)	2.13 (1.07–3.38)	0.082
Triglyceride (mg/dL)	111.0 (66.5–173.8)	114.0 (86.0–154.0)	0.648
HDL cholesterol (mg/dL)	56.0 (47.0–64.0)	57.0 (49.0–69.0)	0.314
LDL cholesterol (mg/dL)	131.0 (110.0–144.3)	125.0 (104.0–138.0)	0.426
FAST score	0.07 (0.04–0.14)	0.47 (0.41–0.59)	<0.001
FIB-4 index	0.94 (0.73–1.43)	1.71 (0.97–1.96)	0.001
Fatty liver index	39.6 (21.0–58.1)	56.1 (35.5–71.1)	0.037
LS (kPa)	4.75 (3.90–5.83)	8.50 (5.80–11.0)	<0.001
CAP (dB/m)	252.5 (219.8–291.3)	298.0 (257.0–331.0)	0.003
MAFLD (n)	52 (52.0%)	17 (68.0%)	0.225
MASLD (n)	37 (37.0%)	16 (64.0%)	0.027
Heavy alcohol drinker (n)	7 (7.0%)	4 (16.0%)	0.305
Positive HBs antigen (n)	2 (2.0%)	0 (0.0%)	1.000
Positive HCV antibody (n)	0 (0.0%)	0 (0.0%)	-

Number or median (range). BMI, body mass index; FAST score, FibroScan–aspartate aminotransferase score; FIB-4 index, fibrosis-4 index; HOMA-IR, homeostasis model assessment of insulin resistance; IQR/med, interquartile range/median; AST, aspartate aminotransferase; ALT, alanine aminotransferase: γGTP, gamma-glutamyl trans-peptidase; HDL, high-density lipoproteins; LDL, low-density lipoproteins; HBs, hepatitis B surface; HCV, hepatitis C virus; MAFLD, metabolic-associated fatty liver disease; MASLD, metabolic dysfunction-associated steatotic liver disease; CAP, controlled attenuation parameter; LS, liver stiffness.

**Table 2:** Correlation among the FAST score, FIB-4 index, fatty liver index, and gut bacteria abundance.

	FAST Score		FIB-4 Index		Fatty Liver Index	
	ρ	*p*-Value	ρ	*p*-Value	ρ	*p*-Value
Phylum						
*Fusobacteria*	0.276	0.002	0.095	0.291	0.121	0.178
Class						
*Fusobacteriia*	0.276	0.002	0.095	0.291	0.121	0.178
Order						
*Fusobacteriales*	0.276	0.002	0.095	0.291	0.121	0.178
Family						
*Fusobacteriaceae*	0.272	0.002	0.096	0.288	0.116	0.197
Genus						
*Fusobacterium*	0.221	0.013	0.040	0.659	0.074	0.415
*Faecalibacterium*	−0.191	0.033	0.074	0.413	−0.218	0.015
*Fusicatenibacter*	−0.160	0.074	−0.113	0.208	0.063	0.485

ρ: Spearman’s rank correlation coefficient; FIB-4 index, fibrosis-4 index; FAST score, FibroScan–aspartate aminotransferase score.

**Table 3:** Risk factors for liver fibrosis after adjusting for the fatty liver index.

	OR	95% CI		*p*-Value
*Fusobacterium*	1.20	1.01	1.43	0.033
*Faecalibacterium*	0.92	0.83	1.03	0.136
*Fusicatenibacter*	0.77	0.61	0.98	0.032

## Data Availability

The data presented in this study are available upon request from the corresponding author. The data are not publicly available because of privacy and ethical restrictions.

## Supplementary Materials

The following supporting information can be downloaded at: https://www.mdpi.com/article/10.3390/ijms241713470/s1.
