# Supplementary material for: Effect of Liver Fibrosis on Oral and Gut Microbiota in the Japanese General Population Determined by Evaluating the FibroScan–Aspartate Aminotransferase Score
Source: Int J Mol Sci. 2023 Aug 30;24(17):13470. doi: 10.3390/ijms241713470 (PMC10487682; doi:10.3390/ijms241713470)
Supplement: Supplementary file 1 [file ijms-24-13470-s001.zip › ijms-2551226-supplementary.pdf]

Supplementary Materials

Supplementary Table S1. Participant characteristics at baseline. Number or median (range).

	FAST< 0.35 (n=812)	FAST≥ 0.35 (n=33)	*p*-value
Sex (male/female)	341:471	21:12	0.012
Age (years)	51.0 (39.0–64.0)	62.0 (44.0–67.0)	0.022
BMI (kg/m2)	22.4 (20.1–24.7)	25.6 (24.2–30.8)	< 0.001
Platelets (×104)	25.7 (22.6–29.9)	23.3 (21.1–28.3)	0.068
Albumin (g/dL)	4.4 (4.2–4.6)	4.4 (4.2–4.6)	0.798
Total bilirubin (mg/dL)	0.8 (0.7–1.0)	0.8 (0.6–1.0)	0.288
AST (U/L)	21.0 (17.0–24.0)	44.0 (38.5–54.0)	< 0.001
ALT (U/L)	17.0 (13.0–24.0)	59.0 (43.0–73.5)	< 0.001
γGTP (U/L)	21.0 (15.0–35.8)	65.0 (46.5–116.0)	< 0.001
Glucose (mg/dL)	91.0 (86.0–99.0)	102.0 (91.0–113.0)	< 0.001
HOMA-IR	1.10 (0.81–1.52)	2.37 (1.26–3.60)	< 0.001
HbA1c (%)	5.6 (5.4–5.8)	6.0 (5.5–6.4)	0.001
Triglycerides (mg/dL)	76.0 (55.0–112.8)	120.0 (87.5–166.5)	< 0.001
HDL cholesterol (mg/dL)	64.0 (54.0–76.0)	55.0 (47.0–65.0)	0.005
LDL cholesterol (mg/dL)	116.5 (96.0–136.8)	122.0 (105.0–137.0)	0.404
Alcohol intake (g/day)	1.3 (0.0–17.6)	4.4 (0.0–30.9)	0.366
FAST score	0.06 (0.03–0.10)	0.49 (0.42–0.62)	< 0.001
FIB-4 index	0.92 (0.64–1.36)	1.67 (0.97–1.96)	< 0.001
Fatty liver index	13.9 (5.92–33.1)	61.9 (49.1–86.9)	< 0.001
LS (kPa)	4.2 (3.5–5.2)	8.6 (6.3–12.3)	< 0.001
CAP (dB/m)	219.0 (185.3–261.0)	305.0 (268.0–338.0)	< 0.001
MAFLD	214 (26.4%)	24 (72.7%)	< 0.001
MASLD	199 (24.5%)	24 (72.7%)	< 0.001
Heavy alcohol drinker	42 (5.2%)	4 (12.1%)	0.182
Positive HBs antigen	8 (1.0%)	0 (0.0%)	0.999
Positive HCV antibody	16 (2.0%)	0 (0.0%)	0.871

BMI, body mass index; FAST score, FibroScan–aspartate aminotransferase score; FIB-4 index, fibrosis-4 index; HOMA-IR, homeostasis model assessment of insulin resistance; IQR/med, interquartile range/median; AST, aspartate aminotransferase; ALT, alanine aminotransferase: γGTP, gamma-glutamyl trans-peptidase; HDL, high-density lipoproteins; LDL, low-density lipoproteins; HBs, hepatitis B surface; HCV, hepatitis C virus; MAFLD, metabolic-associated fatty liver disease; MASLD, metabolic dysfunction-associated steatotic liver disease; CAP, controlled attenuation parameter; LS, liver stiffness
